# Analysis for the Interaction Relationship between Urbanization and Ecological Security: A Case Study in Wuhan City Circle of China

**DOI:** 10.3390/ijerph182413187

**Published:** 2021-12-14

**Authors:** Ji Chai, Zhanqi Wang, Chen Yu

**Affiliations:** 1 Department of Land Resource Management, School of Public Administration, China University of Geosciences (Wuhan), 388 Lumo Road, Hongshan District, Wuhan 430074, China; chaiji_cug@163.com; 2 Safety, Environment, and Technology Supervision Research Institute, Petro China Southwest Oil & Gasfield Company, 12 South Section of Tianfu Avenue, Wuhou Distrct, Chengdu 610041, China; yuchen2015@petrochina.com.cn

**Keywords:** urbanization, ecological security, coupling coordination analysis, impulse analysis

## Abstract

Exploring the interaction relationship between urbanization and ecological security is the key issue to achieve regional sustainable development. This study used coupling coordination model and vector auto-regression model to comprehensively investigate the interaction relationship between urbanization and ecological security in Wuhan City Circle from 2005 to 2018. The results showed that urbanization quality in Wuhan City Circle increased from 0.1818 in 2005 to 0.4355 in 2018, with an average annual increase rate of 10.74%. The ecological security of Wuhan City Circle decreased from 0.4890 in 2005 to 0.4511 in 2015 and increased from 0.4511 in 2015 to 0.4554 in 2018. The degree of coupling coordination between urbanization and ecological security of Wuhan City Circle presented a circle structure with Wuhan as the center and increasing outward. Additionally, the impulse analysis showed that the increase of urbanization had a significant negative impact on ecological security of Wuhan city, Huangshi city, and Xiaogan city. Meanwhile, the ecological security index of Ezhou city, Tianmen city, Huanggang city, Xiantao city, Xianning city, and Qianjiang city were all positive in early stage after the ecological security was impacted by the increase of urbanization. The analysis of historical data and future trends can provide operable recommendations for urbanization development and ecological security protection through cleaner production and efficient use of natural resources from the aspect of coordinated development.

## 1. Introduction

Since the industrial revolution, the world has experienced population explosion and rapid economic development. As a consequence of the urbanization, transition from a traditional rural society dominated by agriculture to a modern urban society has been dominated by industry and services and has proceeded at an unprecedented rate in the past few decades [1]. Cities are the place where modern industries and people are concentrated but also a symbol of human civilization and social progress. The industrial and population agglomeration brought by urbanization have significant effect on promoting regional economic development and human well-being. Meanwhile, urbanization is also a key factor of ecological security by driving multi-scale environmental changes, increasing resource consumption and influencing landscape pattern [2,3]. The growth of urban population and economy will lead to increasing demand for resources from the ecosystem [4]. The urban space expansion can drive landscape pattern and habitat fragmentation, thereby affecting land-use and land-cover change, ecological resilience, and ecosystem services [5]. In addition, the waste emissions from urban areas profoundly influence global climate and biogeochemical cycles [6]. That is to say, urbanization affects the flows of materials, energy, and information and then alters the quality of eco-environment by resources consumption, changing landscape pattern and discharging pollutants, which brings huge challenges to regional ecological security [7].

The interaction relationship between urbanization and environment is currently a major problem that academia and government decision-making departments are generally concerned about and urgently needs to be resolved, and it has become a global strategic issue. In 2005, the International Human Dimensions Program carried out the Urbanization and Global Environmental Change Project and made it a core project of global change research. In 2014, the International Science Union released the “Future Earth 2025 Vision”, which listed urbanization as one of the key research areas. In 2015, the United Nations Sustainable Development Summit put forward 17 Sustainable Development Goals, including sustainable cities and communities and life on land, which proposed to build sustainable cities and protect ecosystems. Meanwhile, the continuous increase in the size and number of cities, especially the emergence of urban agglomerations, and deterioration of ecological environment has become the research hotspot of scholars across the world. Previous studies revealed that the rapid urbanization had a significant impact on regional ecological security [8,9,10]. Some studies have showed that the rural to urban movement had important influence in the increase of non-renewable energy consumption and pollutant discharge through the development of industrial production and population aggregation [11,12,13,14,15,16]. The expansion of urban space occupied plenty of ecological land, such as cultivated land, water bodies, and woodland around the city, which has constituted serious threat to the ecological systems [17,18]. On the evaluation dimension of urbanization, most scholars estimated the quality of urbanization from the perspective of population, economic, and spatial changes [19]. For instance, Fang C.L. and Wang J. explored the ecological risk effect caused by population urbanization, economic urbanization, and spatial urbanization [20]. As to ecological security, some scholars used Pressure-State-Response framework to build ecological security evaluation index system [21,22]. Although Pressure-State-Response framework can reflect the process of urbanization affecting the eco-environment, it cannot clearly reveal the impact mechanism between urbanization and ecological security subsystems. Cui X.G. et al. divided the eco-environment system into resource and environment subsystems to analyze their interaction with urbanization [23]. However, this evaluation system ignored the impact between ecology subsystem and urbanization, such as the changes in ecosystem structure, function, and resilience. In terms of evaluation methods, multi-factor assessment method is the most commonly used. Liu et al. estimated the relationship between urbanization and atmospheric environment security in Jinan City from 1996 to 2004 on the basis of multi-factor assessment method [24]. In comparison, Technique for Order Preference by Similarity to an Ideal Solution (TOPSIS) model can describe the gap between the various evaluation schemes more reasonably, which is not only suitable for comparative analysis between different evaluation object, but also for time series analysis of the same evaluation object. For the interaction relationship between urbanization and eco-environment, most studies focus on the impacts of urbanization on ecological security and the historical change rule of coupling coordination degree between them [25,26,27,28]. Wang Z.B. et al. conducted the coupling coordination degree model and geographic detector model in the Beijing-Tianjin-Hebei urban agglomeration to find out the dominant factors of urbanization and ecology [29]. Qiu M. et al. used grey relationship and decoupling model to study the interaction between urbanization and ecological security in the nine provinces along the Yellow River, China [30]. However, the forecast simulation of interaction trend of urbanization and eco-environment will be an important future research direction [23,31]. At present, few studies have explored the future trend of the relationship between urbanization and ecological security.

Therefore, we attempt to build a system of methodology to clarify the interaction relationship between urbanization and ecological security from the perspective of dynamic change and prediction. Moreover, in this paper, Wuhan City Circle, one of the major urban agglomeration areas in the middle part of China, is selected as an empirical research area. The objectives of this study were to (1) analyze the interaction mechanism between urbanization subsystems (population, economic, and spatial urbanization) and ecological security subsystems (resource consumption, environmental damage, and ecological degradation), (2) assess the level of urbanization and ecological security by TOPSIS model, (3) calculate the coupling coordination degree of urbanization and ecological security to reveal the level of coordinate development, and (4) predict their future changes by Vector Auto-regression (VAR) model. The results can offer novel thoughts for the sustainable management of urban areas and for drafting future ecological security policies.

## 2. Theoretical Mechanism Analysis

### 2.1. The Connotation of Urbanization and Ecological Security

Urbanization system and ecological security system both are complex giant systems combining humanity and nature. On the surface, urbanization is a process in which the rural population continues to converge to the city, but in reality, urbanization also includes changes in the economic structure and land-use patterns [20]. According to the connotation of urbanization and existing research, we divided urbanization into three sub-systems: population urbanization, economic urbanization, and spatial urbanization [32]. The various subsystems of urbanization are interconnected. Population urbanization is the core of the whole system, economic urbanization is the driving force, and spatial urbanization is the representation of spatial pattern change. Ecological security is the condition of ecological environment that ensures that human production, life, and health are not affected [33]. In this paper, ecological security system is composed of three subsystems: resource consumption, environmental damage, and ecological degradation. These three subsystems are all directly affected by the development of urbanization and also reflect the three aspects closely related to ecological security and urbanization.

### 2.2. The Interaction between Urbanization and Ecological Security

**Figure 1:**
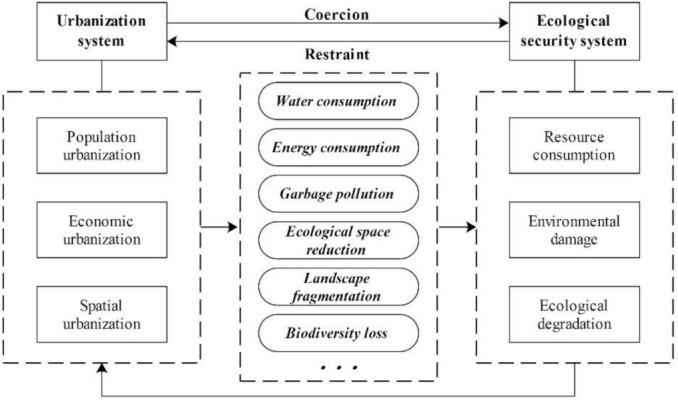
Interaction mechanism between urbanization and ecological security.

Based on the connotation of urbanization and ecological security, we explored the path and mechanism of the interaction between urbanization and ecological security. From the perspective of whole systems, urbanization has a coercive effect on ecological security, and ecological security restricts urbanization in return, as shown in Figure 1.

Accompanied by rapid economic development, population urbanization is mainly reflected in the increase in urban population density and residents’ consumption levels as well as the growing proportion of non-agricultural industry employees. The expansion of the urban population will increase the consumption of natural resources, especially the consumption of water resource. At the same time, it will also produce more domestic garbage and cause environmental pollution. Economic urbanization is mainly reflected in changes in regional production scale and industrial structure. Non-agricultural industries, especially the secondary industry, will increase energy consumption, produce large amounts of industrial wastewater and waste, and cause ecological degradation and environmental pollution. Spatial urbanization provides spatial support for population urbanization and economic urbanization, which is mainly reflected in the change of urban and rural landscapes, increase in urban construction density, and expansion of urban construction land. This process will directly lead to the reduction of enormous high-quality arable land and ecological land around the city, causing fragmentation of the landscape, reduction of ecological space, and damage to biodiversity. Then, it will affect the structure and function of the ecosystem and threaten ecological security. The coercive effect of urbanization on ecological security mainly lies in resource consumption, environmental pollution, and ecological degradation. However, as people’s environmental awareness grows, the decline in the level of ecological security can also restrict the development of urbanization. City managers set the upper limit of carrying capacity to constrain the consumption intensity of human activities on nature and control the scale of the city. Meanwhile, more environmental protection funds should be invested to develop cleaner production technologies to reduce resource consumption and environmental pollution. City managers can also prevent urban land from encroaching on the ecological space by strictly controlling the growth of urban construction land, further optimizing the layout of industrial land, and improving the level of land conservation and intensive use. In summary, the core of this interaction relationship is the contradiction between the pressure of human activities and the supporting capacity of environment.

## 3. Study Area and Data Sources

### 3.1. Study Area

**Figure 2:**
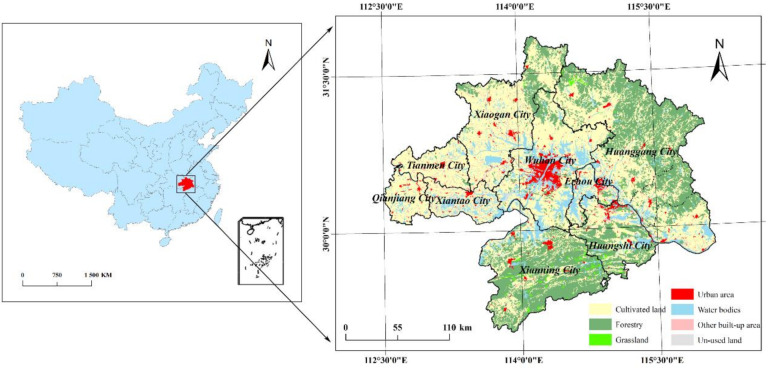
Location of the Wuhan City Circle and its land-use status of 2018.

Wuhan City Circle, also as known as Wuhan “1+8” City Circle, refers to an urban agglomeration centered on Wuhan city, which is the largest city in central China and the capital of Hubei province, consisting of eight large- and medium-sized cities around it, including Huangshi city, Ezhou city, Huanggang city, Xiaogan city, Xianning city, Xiantao city, Tianmen city, and Qianjiang city. Wuhan City Circle is located in middle reaches of Yangtze River. The major geographic types of Wuhan City Circle are plains and hills. The Wuhan City Circle, which is less than one-third area of Hubei Province, has more than half of the Hubei’s population and more than 60% of the total GDP. It is one of the largest urban clusters in central China. Based on the data of Resource and Environment Science and Data Center of China, the location of Wuhan City Circle and its land-use status of 2018 are shown in Figure 2.

Wuhan City Circle is not only the core area of Hubei’s economic development but also an important growth pole for the rise of middle part of China. In 2004, the Hubei Provincial Government officially started the integrated construction of Wuhan City Circle. Since then, the Wuhan City Circle has entered a period of rapid development. After more than ten years of development, the coordinated development of Wuhan City Circle has made obvious progress. In 2018, the GDP of Wuhan City Circle reached 2.50 trillion yuan, of which the added value of the tertiary industry accounts for 52.01% of the total GDP [34]. The permanent population of Wuhan City Circle in 2018 was 31.80 million, and the urbanization rate was 63.74%, which is higher than the average of Hubei Province [34]. Meanwhile, the forest area of Wuhan City Circle is 30.11% of total area, and the area of water bodies is 10.93% of total area in 2018. Due to the abundant water and wetland resources in the area, Wuhan City Circle also is an important ecological function reserve in China, which is significant to optimize the pattern of regional ecological security. Therefore, exploring the interaction relationship of urbanization and ecological security in Wuhan City Circle has important practical significance for realizing regional sustainable development, and it also provides reference for the sustainable development of other areas.

### 3.2. Data Sources

The research period selected for this study is 2005–2018. Since Hubei Province proposed the construction of Wuhan City Circle in 2004, nine cities in the Wuhan City Circle have entered a period of rapid development. In this paper, we selected the years of 2005, 2010, 2015, and 2018 as the study time point to show their spatial-temporal changes. The land-use data used in this paper come from the land-use/land-cover remote sensing monitoring data of China based on Landsat-TM/ETM and Landsat 8 remote sensing images provided by Resource and Environment Science and Data Center of Chinese Academy of Sciences [35]. The type of land-use data used in this paper is raster data, with 30 meters’ precision. The ecological environment data for 2005–2018 come from the Hubei Provincial Ecological Environment Bulletin [36]. The urban economic and social data for 2005–2018 come from the China Urban Construction Statistical Yearbook and the Hubei Provincial Statistical Yearbook [37,38].

## 4. Methods

### 4.1. Comprehensive Evaluation of Urbanization and Ecological Security

#### 4.1.1. Data Standardization and Indicator Empowerment

Due to the dimensional difference of the evaluation indicators and their positive and negative effects on the corresponding system, we adopt the method of extreme value standardization to process the original data dimensionless, which is convenient for comprehensive calculation and comparative analysis.

The formula for positive and negative indicators are given as:

(1) $$b_{ij}^{*}=\frac{b_{ij}-\min\left(b_{ij}\right)}{max\left(b_{ij}\right)-\min\left(b_{ij}\right)}$$

(2) $$b_{ij}^{*}=\frac{max\left(b_{ij}\right)-b_{ij}}{max\left(b_{ij}\right)-\min\left(b_{ij}\right)}$$

where $b_{ij}^{*}$ represents standardized value of *j*-th indicator; $max\left(b_{ij}\right)$ and $\min\left(b_{ij}\right)$ refer, respectively, to the maximum and minimum value of original data of *j*-th indicator; *i* refers the sample number of *j*-th indicator.

In the comprehensive evaluation of urbanization and ecological security, to reduce the interference of subjective factors and reflect the utility value of the index information entropy, this paper intends to use the entropy weight method to calculate the weight coefficient of each index.

The formula of comentropy of indicator is given as:

(3) $$e_{j}=-k\overset{n}{\underset{j=1}{\sum }}f_{ij}\;\ln f_{ij}$$

(4) $$k=\frac{1}{\ln i}$$

where $e_{j}$ represents comentropy of *j*-th indicator, k represents adjustment coefficient, and $f_{ij}$ represents percentage of $b_{ij}^{*}$ of total standardized value.

The formula of indicator difference coefficient is given as:

(5) $$h_{j}=1-e_{j}$$

where $h_{j}$ refers to the *j*-th indicator difference coefficient.

The formula of indicator weight is given as:

(6) $$\omega_{j}=\frac{h_{j}}{\sum_{j=1}^{n}h_{j}}$$

where $\omega_{j}$ represents weight of *j*-th indicator, $\omega_{j}\in \left[0,1\right]$, and $\overset{n}{\underset{j=1}{\sum }}\omega_{j}=1$.

#### 4.1.2. Urbanization and Ecological Security Evaluation Index System

For accurately evaluating the urbanization and ecological security of Wuhan City Circle, we used frequency statistics and expert consultation methods to determine the evaluation indicators system based on the existing research achievements and characteristics of the study area. The urbanization and ecological security evaluation indicators system established in this study is shown in Table 1. The entropy weight of each indicator is calculated according to Formula (1) to (6) and the value of each indicator.

The urbanization evaluation index system is composed of 10 indicators, including three dimensions: population urbanization, economic urbanization, and spatial urbanization. Population urbanization reflects the transition of rural population to urban population. Economic urbanization reflects the situation of urban economic development and industrial transformation. Spatial urbanization reflects the transformation of urban land and the evolution of urban spatial structure. The ecological security evaluation index system is divided into three dimensions of resource, environment, and ecology by 11 indicators. Resource reflects the consumption level and utilization efficiency of urban resources. Environment reflects the environmental quality and the comfort of living environment of the city. Ecology reflects the urban landscape ecological pattern and ecosystem function.

The description of common indicators in the indicator system will not be repeated, and the three indicators of patch density, ecosystem service value, and ecological resilience index will be explained mainly. Patch density is an important indicator to describe the fragmentation of the landscape. The formula of patch density is given as:

(7) $$PD=\frac{N}{A}$$

where N refers to the number of patch types in the area, and A refers to the total area.

Ecosystem service value is the products and services obtained through an ecosystem for survival and development of mankind. The calculation of ecosystem service value refer to previous studies of Costanza R. et al. and Xie G.D. et al. [39,40]. According to the correspondence of the land-use type and ecosystem type, we get the ecosystem service value equivalent per unit area of this paper from previous research results, as shown in Table 2. It represents the importance value of different type of ecosystem services relative to the value of farmland food production.

The formula of economic value of a unit ecosystem service equivalent value is calculated by:

(8) $$Ea=\frac{1}{7}\times \sum_{i=1}^{n}\frac{s_{i}p_{i}y_{i}}{S}$$

where Ea refers to the economic value of a unit ecosystem service equivalent value, $s_{i}$ represents cultivated area of *i*-th grain, $p_{i}$ refers to average price of *i*-th grain, $y_{i}$ refers to the per unit area yield of *i*-th grain, and S refers to the total cultivated area of food crops.

The formula of ecosystem service value is given as:

(9) $$ESV=Ea\times Ev\times e_{j}$$

where ESV refers to the ecosystem service value, $e_{j}$ refers to the area of *j*-th ecosystem type, and Ev refers to the ecosystem service equivalent value per unit area.

The ecological resilience index reflects the contribution and effect of different land on ecological restoration. With reference to existing research [41], we assign values to the ecological resilience of each land-use type to obtain the regional ecological resilience index, as shown in Table 3.

The formula of ecological resilience index is given as:

(10) $$Er=\overset{n}{\underset{i=1}{\sum }}\frac{a_{i}R_{i}}{A}$$

where refers to the ecological resilience index, $a_{i}$ refers to the area of *i*-th land-use type, $R_{i}$ refers to the ecological resilience of *i*-th land-use type, and A refers to the total area of region.

#### 4.1.3. TOPSIS Model

TOPSIS model is a common method in multi-objective decision analysis, which was first proposed by Hwang C.L. and Yoon K. in 1981. In this paper, we introduced TOPSIS model to assess the pros and cons of indicators through calculating the distance between indicators and positive/negative ideal solutions.

Based on the results of data standardization and indicator empowerment, the weighted decision matrix is calculated as follows:

(11) $$R=\left[\begin{array}{cccc} b_{11}^{*}\times \omega_{1} & b_{12}^{*}\times \omega_{2} & \cdots & b_{1j}^{*}\times \omega_{j}\\ b_{21}^{*}\times \omega_{1} & b_{22}^{*}\times \omega_{2} & \cdots & b_{2j}^{*}\times \omega_{j}\\ \cdots & \cdots & \cdots & \cdots \\ b_{n1}^{*}\times \omega_{1} & b_{n2}^{*}\times \omega_{2} & \cdots & b_{ij}^{*}\times \omega_{j} \end{array}\right]$$

where $b_{ij}^{*}$ represents the standardized value, and $\omega_{j}$ represents the weight.

The formula for positive and negative ideal solutions are given as:

(12) $$R^{+}=\left\{\underset{1\leq j\leq m}{\max}b_{ij}^{*}|j=1,2,\;\cdots ,j\right\}=\left\{b_{1}^{*+},b_{2}^{*+},\cdots ,b_{j}^{*+}\right\}$$

(13) $$R^{-}=\left\{\underset{1\leq j\leq m}{\min}b_{ij}^{*}|j=1,2,\;\cdots ,j\right\}=\left\{b_{1}^{*-},b_{2}^{*-},\cdots ,b_{j}^{*-}\right\}$$

where $R^{+}$ and $R^{-}$ represent positive and negative ideal solutions, and $b_{j}^{*+}$ and $b_{j}^{*-}$ represent the positive and negative ideal values of *j*-th indicator.

The close degree of indicator is calculated by:

(14) $$D_{i}^{+}=\sqrt{\sum_{j=1}^{m}{\left(b_{j}^{*+}-b_{ij}^{*}\right)}^{2}}$$

(15) $$D_{i}^{-}=\sqrt{\sum_{j=1}^{m}{\left(b_{j}^{*-}-b_{ij}^{*}\right)}^{2}}$$

(16) $$T_{i}=\frac{D_{i}^{-}}{D_{i}^{+}+D_{i}^{-}}$$

where $D_{i}^{+}$ refers to Euclidean Distance between the *j*-th indicator in *i*-th year and $R^{+}$, $D_{i}^{-}$ refers to euclidean distance between the *j*-th indicator in *i*-th year, and $R^{-}$, $T_{i}$ refers to the close degree of *j*-th indicator, $T_{i}\in \left[0,1\right]$.

### 4.2. Coupling Coordination Model

To quantitative analyze the dynamic interaction of urbanization and ecological security, coupling coordination model is adopted in this paper. Coupling degree is used to explore the interaction effect between systems. Moreover, coupling coordination degree can reveal the level of benign coupling in the coupling interaction relationship.

The formula of coupling degree is given as:

(17) $$C=\frac{U_{i}^{2}\times E_{i}^{2}}{{\left(\frac{U_{i}+E_{i}}{2}\right)}^{4}}$$

where C represents coupling degree, $U_{i}$ refers to the quality of urbanization, and $E_{i}$ refers to the level of ecological security.

The formula of coupling coordination degree is given as:

(18) $$D={\left[C\times \left(\mu U_{i}+\omega E_{i}\right)\right]}^{\frac{1}{2}}$$

where D refers to the coupling coordination degree, and μ and ω represent weight coefficients: μ=0.5, ω=0.5 [27].

Meanwhile, for better showing the changes of coupling coordination level, the coupling coordination degree of urbanization and ecological security is divided into four categories and 12 subcategories.

### 4.3. Vector Auto-Regression Model

Vector auto-regression (VAR) model is an econometric analysis model based on the statistical properties of data proposed by Christopher A.S. in 1980 [42]. VAR model is often adopted to investigate the dynamic impact of random disturbance items on system variables, reveal the impact of variable shocks on the explained variables, and predict the dynamic effects between two systems that affect each other.

The mathematical expression of VAR model is given as:

(19) $$y_{t}=c+\overset{p}{\underset{j=1}{\sum }}A_{j}y_{t-j}+\mu_{t}\;$$

where $y_{t}$ refers to time series vector, c refers to constant term, p refers to auto-regressive lag order, $A_{j}$ refers to time series coefficient matrix, and $\mu_{t}$ refers to white noise sequence vector.

In this paper, we established a two-variable VAR model composed of urbanization quality index and ecological security level in Wuhan City Circle. Then, stationary test and impulse-response analysis were used for dynamic analysis of the interaction relationship of them. The Augmented Dickey–Fuller test was used for stationary test to determine whether the variable data of the time series was stationary data so as to avoid the phenomenon of pseudo-regression in the VAR model. Impulse-response analysis was used to characterize the dynamic impact of urbanization on ecological security and to predict their changes. Impulse-response analysis can better show the influence of a standard deviation shock applied to the random error term on the current and future values of the variable.

## 5. Results

### 5.1. Variation Tendency of Urbanization in Wuhan City Circle

**Figure 3:**
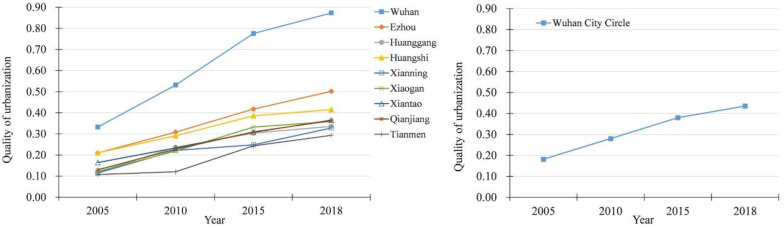
The changes of urbanization quality in Wuhan City Circle.

The variation tendency of the quality of urbanization of cities and region in Wuhan City Circle from 2005 to 2018 are shown in Figure 3, using TOPSIS model.

As shown in Figure 3, the quality of urbanization of Wuhan City Circle increased from 0.1818 in 2005 to 0.4355 in 2018, with an annual mean growth rate of 10.74%. It reflected that the urbanization of Wuhan City Circle was showing a steady upward trend during 2005 to 2018 from the perspective of the region as a whole. The urbanization quality of all cities in Wuhan City Circle also increased to varied extent. This showed that the construction of Wuhan City Circle had achieved remarkable results, and the level of urbanization development had been continuously improved. However, from the perspective of spatial pattern, the development differences among the cities were expanding from 2005 to 2018. From line chart, the urbanization of the Wuhan City Circle showed the remarkable characteristics of Wuhan’s single-core leader. At the same time, the difference in urbanization quality between Wuhan and other cities was becoming more and more obvious. In 2005, the urbanization quality of Wuhan city was 0.3324, while the average level of the other eight cities was 0.1472. In 2018, the urbanization quality of Wuhan city was 0.8727, while the average level of the other eight cities was 0.5028. The gap between Wuhan city and other eight cities widened by 1.7 times from 2005 to 2018. The results revealed that although the urbanization level of each city had been improved, the uneven development within Wuhan City Circle was increasing. In addition, the urbanization development level of the other eight cities were also different in study period. From 2005 to 2018, the quality of urbanization in Ezhou city and Huangshi city were relatively high. In addition, the urbanization quality of the remaining six cities were basically the same, increasing from about 0.1 to about 0.3. The quality of urbanization of Ezhou city and Huangshi city increased from 0.2100 and 0.2102 in 2005 to 0.5016 and 0.4152 in 2018. This is because Ezhou city and Huangshi city have advantages in both their location and industrial resources. Ezhou city is a national logistics hub city, with convenient shipping and abundant water resources. Meanwhile, Huangshi city has rich mineral resources, such as iron ore and copper ore, which greatly boosted its mining industry and urbanization.

### 5.2. Variation Tendency of Ecological Security in Wuhan City Circle

**Figure 4:**
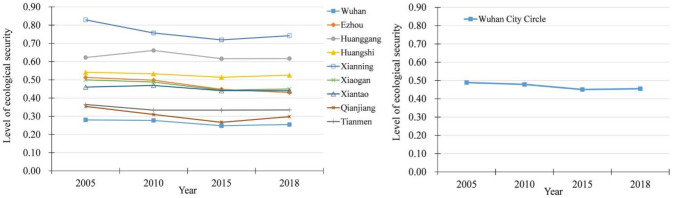
The changes of ecological security level in Wuhan City Circle.

According to evaluation indicators system and TOPSIS model, we evaluated the ecological security level of cities scale and region scale in Wuhan City Circle from 2005 to 2018. The results are shown in Figure 4.

From Figure 4, we can see that the variation of overall ecological security of Wuhan City Circle can be divided into two stages. From 2005 to 2015, there was a stage of gradual decline, which kept decreasing from 0.4890 in 2005 to 0.4511 in 2015. Then, the ecological security of Wuhan City Circle entered a slow growth stage during next three years between 2015 and 2018, which increased from 0.4511 in 2015 to 0.4554 in 2018. It revealed that the rapid urbanization and large-scale construction pose a greater threat to ecological security in Wuhan City Circle from 2005 to 2015. After 2015, central government proposed that the Yangtze River Economic Belt should implement a development path of ecological priority and green development. Since then, in the urban planning and construction of the Wuhan City Circle, more considerations of ecological protection had emerged. Although it was in the period of rapid urbanization, the industrial economy and infrastructure construction had continued to advance, and the ecological security level of the Wuhan City Circle had begun to improve slightly. From the perspective of spatial pattern, the development of urbanization and ecological security in the Wuhan City Circle had formed a clear mismatch relationship. Cities with rapid urbanization development have a lower level of ecological security, while cities with slower urbanization development have a higher level of ecological security. As a city with the highest urbanization quality in Wuhan City Circle, the ecological security of Wuhan city had been at the lowest level of whole region during the study period, which was 0.2800 in 2005 and 0.2547 in 2018. Moreover, the level of ecological security of Xianning city, which was 0.8290 in 2005 and 0.7422 in 2018, was the highest in the Wuhan City Circle, while its urbanization quality was at a low level in the entire region. This phenomenon confirms the urbanization has a coercive effect on ecological security, and ecological security restricts the development of urbanization to some extent. It also indicates the ecological security level of Wuhan City Circle is very likely to be reduced again if the scale and development intensity of urban expansion are not strictly controlled in the future urbanization.

### 5.3. Coupling Coordination Analysis of Urbanization and Ecological Security

**Figure 5:**
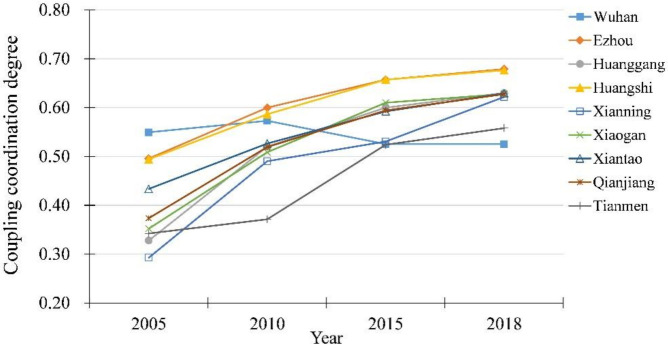
The changes of coupling coordination degree of urbanization and ecological security.

**Figure 6:**
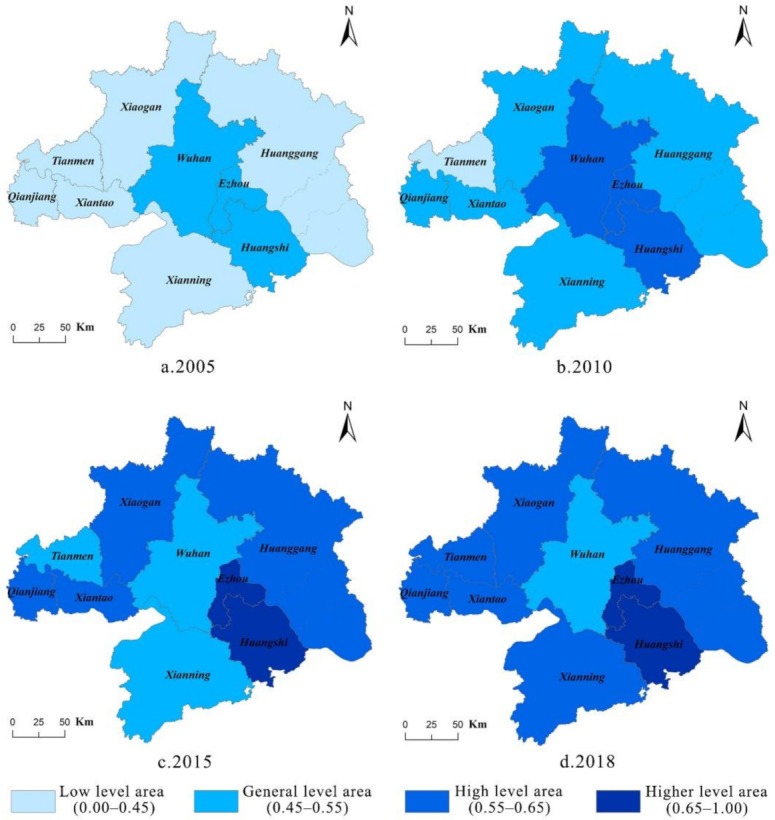
Spatial distribution of the coupling coordination degree.

According to Formula (17) and (18), we calculated the coupling coordination degree of urbanization and ecological security in Wuhan City Circle for achieving their coordinated development, as shown in Figure 5 and Figure 6.

In Figure 5, coupling coordination degree of each city fluctuate in different patterns. From 2005 to 2018, the degree of coupling coordination of Wuhan city first increased from 0.5493 in 2005 to 0.5730 in 2010, then dropped to 0.5252 in 2018. Wuhan city had the highest level of coupling coordination in Wuhan City Circle at 2005 and lowest at 2018. The coupling coordination degree of Ezhou city and Huangshi city were always at a higher level in Wuhan City Circle, which reached above 0.65 in 2015 and 2018. During study period, Xianning City had the biggest percentage jump of coupling coordination degree, which increased by 112.28% from 0.2928 to 0.6215. Similarly, Huanggang city and Xiaogan city increased, respectively, by 92.11% and 78.28%, which showed a trend of substantial growth. As for three cities in the west of Wuhan City Circle, the coupling coordination degree of Qianjiang city and Tianmen city increased, respectively, from 0.3736 and 0.3422 in 2005 to 0.6273 and 0.5580 in 2018. Additionally, the change of coupling coordination degree in Xiantao City was relatively flat, of which growth rate was 45.15%.

From the perspective of spatial distribution, the coupling coordination degree of Wuhan City Circle presented a circle structure with Wuhan as the center and increasing outward. This was related to the development positioning and ecological endowment of the cities in the Wuhan urban circle. Although the level of urbanization in Wuhan was significantly higher than that of other cities, the problems of resource waste, environmental pollution, and ecological degradation in Wuhan were more serious, causing serious misalignment in the development of urbanization and ecological security level. In the latter part of the study period, Wuhan City used policy interventions and planning methods to restrict and guide the city’s development, which stopped the downward trend of coupling and coordination. Conversely, most of the cities in the periphery of Wuhan City Circle improved their coupling and coordination degree to a certain extent during the study period. Because of good ecological background conditions and sufficient ecological space, the ecological security of these cities had a strong carrying capacity for the stress of urbanization.

**Figure 7:**
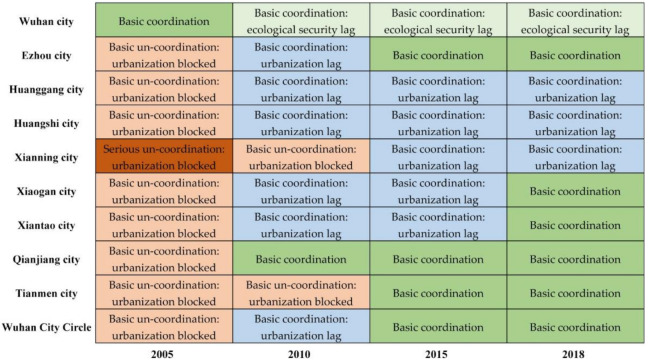
The classification of coupling coordination degree.

Based on the classification standard of Table 4, we divided the coupling coordination relationship into several types as shown in Figure 7.

During 2005 to 2018, the coupling coordination level of Wuhan city showed a downward trend. Wuhan city, the center of this urban agglomeration, is the only city whose coupling coordination types regressed during the study period. Wuhan city had always been in high-speed development period of urbanization, and its population, spatial, and economic urbanization levels were in a higher level among Wuhan City Circle. However, the growth of urban population, rapid industrialization, and urban expansion caused a serious imbalance in the ratio of construction land to ecological space in Wuhan, which put greater pressure on resource consumption and environmental protection. At the same time, the sharp decline of cultivated land and ecosystem services value led to uncoordinated development of urbanization and ecological security in Wuhan city.

In the eastern part of Wuhan City Circle, the type of coupling coordination in Ezhou City changed from “basic un-coordination: urbanization blocked” to “basic coordination”, showing the characteristics of a transition from uncoordinated to coordinated development. In 2005, the quality of urbanization in Ezhou city lagged behind the level of ecological security. Subsequently, relying on location advantages to vigorously develop high-tech industries, Ezhou city continued to expand, and the economy achieved rapid development. The restrictive effect of ecological security on urbanization gradually weakened, and the relationship between them reached a state of basic coordination. The coupling coordination level of Huanggang city and Huangshi city improved from “basic un-coordination: urbanization blocked” to “basic coordination: urbanization lag”. The reason was that urbanization development level of these two cities in the eastern mountainous area, especially the spatial urbanization, were relatively low. In the southern part of Wuhan City Circle, the type of coupling coordination in Xianning City changed from “serous un-coordination: urbanization blocked” to “basic coordination: urbanization lag”. Although the coupling coordination level of Xianning city was greatly improved during the research period, it is still necessary to further improve the quality of urbanization while ensuring ecological security in the future. In the western area, the type of coupling coordination in Tianmen city, Xiantao city, Xiaogan City, and Qianjiang city changed from “basic un-coordination: urbanization blocked” to “basic coordination”. Most of these cities are located in the Jianghan Plain. They are important rice-, cotton-, and rapeseed-planting bases in China, providing a strong material guarantee for national food security. At the beginning of study period, Tianmen city, Xiantao city, Xiaogan City, and Qianjiang city were less affected by Wuhan’s radiation driving effect, and their urbanization development was relatively slow. As the concept of Wuhan City Circle was put forward, these cities took the initiative to introduce high-quality industrial projects and continue to promote the transfer of rural labor, which has greatly increased the speed of urbanization. In the end, the coupling coordination level of these cities reached the state of basic coordination.

### 5.4. Analysis of Dynamic Effects between Urbanization and Ecological Security in Wuhan City Circle

Based on VAR model, we used the results of urbanization and ecological security of Wuhan City Circle from 2005 to 2018 as a sample to reveal dynamic effects between them. Since VAR model requires data of each indicator to be stable, we use the logarithmic value of each variable in the analysis. The advantage of logarithmic processing is that it does not change the change characteristics of the original data, and the logarithmic data are more stable than the original data. With the help of Eviews8.0 software, we used the Augmented Dickey–Fuller (ADF) test method to test the stationary of the urbanization quality and ecological security level of Wuhan City Circle and its first-order difference sequence.

**Figure 8:**
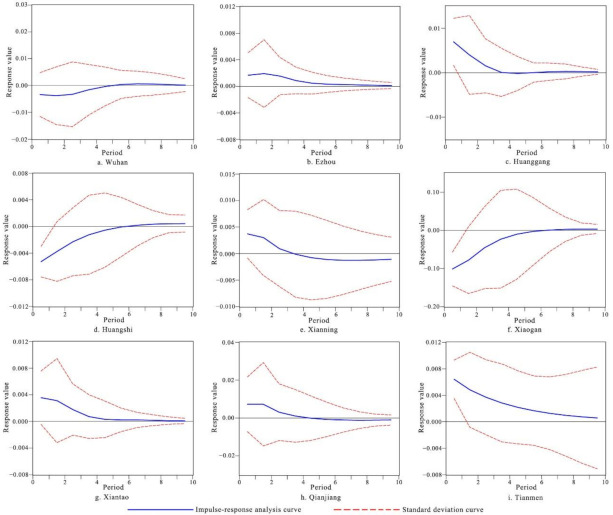
Response of ecological security to urbanization in Wuhan City Circle.

In Table 5, results showed that after the first-order difference processing of each variable’s data, the T-value was less than the critical value at the 10% significance level; that is, the DlnU and DlnE of each city after the first-order difference were stationary series. Therefore, this article established a VAR model for DlnU and DlnE and through impulse-response analysis to investigate the dynamic effects of urbanization and ecological security. The results of impulse-response analysis are shown in Figure 8.

According to the impulse response of ecological security to urbanization, the ecological security index of Wuhan city, Huangshi city, and Xiaogan city were all negative in first period after the ecological security was impacted by the increase of urbanization. Moreover, the ecological security index of these cities gradually stabilized after the sixth period. This showed that under the current development model of high-capital investment and energy consumption, the rapid increase of urbanization in the future will further inhibit the improvement of urban ecological security. Therefore, getting rid of the development model driven by factors, such as resource input and land expansion, is still the focus of the coordinated development for these cities in the future. The ecological security index of Ezhou city, Tianmen city, Huanggang city, Xiantao city, Xianning city, and Qianjiang city were all positive in the first period after the ecological security was impacted by the increase of urbanization. The ecological security index of most cities also remained positive during the whole period, except for Xianning city and Qianjiang city. This revealed that the urbanization and ecological security of these cities were showing a trend of coordinated development, which was an important path to achieve sustainable development. Results of ecological security assessment showed that the ecological security level of most of these cities had been improved in 2015 to 2018. For example, the ecological security index of Huanggang city and Xianning city increased, respectively, from 0.6158 to 0.6165, and 0.7188 to 0.7422 in 2015 to 2018. Since the Chinese government vigorously promoted the construction of ecological civilization in 2012, many cities changed their traditional development thinking that focuses on the expansion of economic aggregates. City managers carried out industrial layout and resource allocation through urban planning and have made positive progress in ecological protection, resources conservation, and emission reduction. The ecological security level of Xianning city and Qianjiang city showed a volatility trend that first decreased and then increased. It illustrates that although growth of urbanization in the early stage could enhance ecological security, if they do not adhere to develop cleaner production and intensive use of land, urbanization in the later stage will become a threat to ecological security.

## 6. Discussion

### 6.1. The Interaction Mechanism between Urbanization and Ecological Security

The coordinated improvement of urbanization and ecological security is one of the core contents of achieving sustainable development. In this paper, we tried to theoretically analyze the interaction mechanism between urbanization subsystems and ecological security subsystems and take the Wuhan City Circle as an example to quantitatively analyze and forecast interaction relationship between them. Some scholars have also carried out studies on the relationship of urbanization and ecological environment. In the evaluation of ecological security, most of the existing studies regarded ecological security as a whole and used the PSR model to analyze the pressure on ecological security caused by changes of urbanization subsystems, the state of the ecological security system, and the measures taken to maintain ecological security [21,22]. Although such a theoretical analysis framework can well reflect the process of urbanization affecting ecological security, it cannot clearly reveal the changes within the ecological security subsystems. Therefore, different from the existing studies, this paper divided ecological security into the three subsystems of resource consumption, environmental damage, and ecological degradation for clarifying the coercing and coercing and restricting effects paths between these three subsystems and the subsystems of population, economic, and spatial urbanization.

After qualitatively analysis of the interaction between urbanization and ecological security, this study utilized the coupling coordination model and VAR model to verify and predict the correlation between urbanization and ecological security. In existing studies, the analysis of coupling coordination usually includes the changing laws of historical data, such as historical changes of coupling degree, coupling coordination degree, and coupling coordination type [25,26,27,28,29,30]. However, the characteristics of effects of future urbanization changes on ecological security are usually neglected [23,31]. The dynamic effects between urbanization and ecological security reflects the trend of ecological security level under the current urbanization development model. Especially under the background of rapid progress of urbanization, urban expansion in China is still occurring. It is necessity to forecast simulation of interaction trend of urbanization and ecological security for regional sustainable development.

### 6.2. Policy Implications of Coupling Coordination Analysis and Impulse Analysis

Due to the coercive effect of urbanization on ecological security and the restrictive effect of ecological security on urbanization, it is very difficult to realize the coordinated development of the two under the traditional development model in the past. By adopting a case study of Wuhan City Circle, this paper used the coupling coordination analysis and impulse analysis to quantitatively analyze the changing laws of historical data and future trends of urbanization and ecological security. The results can provide policy implications for coordinated development of urbanization and ecological security in the Wuhan city circle.

According to the results, since cities in Wuhan City Circle development varies, differentiated urban development and ecological protection mechanisms should be established according to the assessment results. Wuhan city should slow down the speed of urbanization and improve the ecological environment in order to achieve their coordinated development. Policy makers can reduce the occupation of agricultural space and ecological space by promoting the conservation and intensive use of urban land, promoting resources conservation, reducing wastes discharge to decrease the consumption of non-renewable resources, and developing cleaner production. Additionally, more environmental protection funds should be invested to develop cleaner production technologies to reduce resource consumption and environmental pollution in Wuhan city. Huanggang city, Huangshi city, and Xianning city should vigorously improve the level of economic urbanization while strengthening ecological protection. These cities can take advantage of the implementation of strategies, such as the Yangtze River Economic Belt and Wuhan City Circle, and promote economic improvement through the development of emerging industries, such as tourism, biotechnology, and new energy industries. Other cities in the Wuhan City Circle should maintain the current development model to continuously improve the quality of urbanization and strengthen ecological protection for achieving sustainable economic and social development.

In order to achieve regional coordinated development of Wuhan City Circle, cities should strengthen cooperation in urban development and ecological protection. In the development of urbanization, each city should form a spatial pattern of inter-regional industrial division and cooperation based on their respective development advantages and potentials. By optimizing the industrial space layout and rationally distributing element resources, Wuhan City Circle should build a modern industrial system with complementary advantages and close collaboration to achieve high-quality urbanization development. For the protection of ecological security, Wuhan City Circle should coordinate to strengthen environmental protection and promote green recycling and low-carbon development. Especially in air pollution control, water pollution control, and wetland protection, each city should actively take effective measures.

### 6.3. Limitations of the Present Study

Several limitations of present study need to be solved in future studies. First, the quantification of urbanization can be analyzed from more aspects. In this study, only the population, economy, and space are considered in urbanization evaluation; other social aspects, such as urban medical condition, urban employment rate, and urban road area per capita, can be combined in future studies. Second, the evaluation index system of ecological security can be improved. The main thought of this study is to explore the interaction relationship between urbanization and ecological security. Therefore, we mainly selected the evaluation indicators that are significantly affected by urbanization. Future research can improve the evaluation index system of ecological security from natural background factors and other aspects. Third, this study focused on analyzing the dynamic effects of the urbanization and ecological security from the perspective of ecological security’s response to urbanization. The coupling coordination level of urbanization and ecological security can be predicted in the following studies by adopting analytical models, like spatial Markov chain method. Beyond that, in order to better guide the healthy development of urbanization and environment, further studies should pay close attention to apply spatial autocorrelation analysis in coupling coordination spatial pattern of urbanization and ecological security.

## 7. Conclusions

The interaction relationship of urbanization and ecological security were examined in this paper using TOPSIS model, coupling coordination model, and VAR model. As an important ecological protection and urban growth pole in the Yangtze River Economic Zone, sustainable development of Wuhan City Circle has important reference value for other urban agglomeration areas. The conclusions are summarized as follows:

This study demonstrated the interaction mechanism between urbanization and ecological security by a theoretical analysis framework. Specifically, the coercing and restricting effects paths between urbanization and ecological security subsystems were clarified to provide a theoretical basis for this study. The development of urbanization is manifested in urban population growth, economic structure changes, and urban spatial expansion. Along with this process, the increase in resource consumption, the destruction of environmental pollution, and the degradation of the ecosystem have all seriously affected ecological security. Meanwhile, as people’s environmental awareness grows, the continuous deterioration of the ecological environment will also prompt the government to take measures to limit the disorderly development of urbanization.

This study built an urbanization and ecological security evaluation index system and adopted the TOPSIS model to comprehensively evaluate the urbanization and ecological security. The evaluation index system based on the interaction mechanism between urbanization and ecological security can clearly reveal the impact between their subsystems. TOPSIS model has the advantage of describing the gap between the various evaluation schemes more reasonably. The evaluation results also showed that the evaluation system constructed in this paper had better revealed the urbanization quality and ecological security level of the cities in Wuhan City Circle.

In order to accurately reveal the interaction between urbanization and ecological security, this paper used a coupling coordination model and VAR model to quantitatively investigate the level of coupling coordination development and dynamic effects of the two. Coupling coordination degree can represent the level of benign coupling in the coupling interaction relationship, which is a common method to study the relationship between systems. VAR model was introduced to reveal the impact of urbanization shocks on the ecological security and predict the dynamic effects between two systems. The results of coupling coordination analysis and impulse analysis indicated the coordinated development degree and future trends of urbanization and ecological security in Wuhan City Circle, which provide support for the sustainable management of urban areas and for drafting future ecological security policies.

Additionally, there are several limitations of present study need to be solved in future studies. Due to the limit of data sources, the evaluation index system of urbanization and ecological security can be improved by adding social urbanization indicators and natural background factors. In addition, future research can use other forecasting methods and spatial analysis methods to further explore the coordinated development level of urbanization and ecological security, such as spatial Markov chain method and spatial autocorrelation analysis. Moreover, the methods of this study can also be used to conduct empirical research in other urban agglomerations or larger-scale research areas to provide comprehensive and targeted support for urban management and ecological protection for other regions with rapid urbanization.

## Figures and Tables

**Table 1:** The urbanization and ecological security evaluation indicators system.

Target Layer	Subsystem Layer and Weight	Indicator Layer	Entropy Weight	Indicator Type
Urbanization	Population urbanization (0.2234)	Proportion of non-agricultural population (%)	0.1327	+
		Urban population density (people/km^2^)	0.0907	+
	Economic urbanization (0.3590)	Per capita GDP (yuan/people)	0.0929	+
		The proportion of the secondary and tertiary industries in GDP (%)	0.0608	+
		Per capita total retail sales of social consumer goods (yuan/people)	0.0905	+
		Per capita fiscal revenue (yuan/people)	0.1148	+
	Spatial urbanization (0.4176)	The proportion of built-up area to total urban area (%)	0.1026	+
		Per capita urban road area (m^2^/people)	0.0571	+
		Per land output value of secondary and tertiary industries (10,000 yuan/km^2^)	0.1321	+
		Per land fixed asset investment (10,000 yuan/km^2^)	0.1258	+
Ecological security	Resource (0.2804)	Per capita daily domestic water consumption (L/people·day)	0.0387	−
		Change rate of unit GDP energy consumption (%)	0.0281	−
		Per capita built-up area (m^2^/people)	0.1463	−
		Unit GDP water consumption (t/10,000 yuan)	0.0673	−
	Environment (0.2446)	Wastewater discharge intensity (t/km^2^)	0.0748	−
		Green coverage rate in built-up area (%)	0.0310	−
		Per capita park green area (m^2^/people)	0.0526	+
		Good air quality rate (%)	0.0862	+
	Ecology (0.4750)	Patch density	0.0762	−
		Ecosystem service value (yuan)	0.1956	+
		Ecological resilience index	0.2032	+

**Table 2:** Ecosystem service equivalent value per unit area.

Ecosystem Type	Equivalent Value	Ecosystem Type	Equivalent Value
Paddy land	3.89	Grassland	12.06
Dry land	4.01	Water bodies	125.61
Shrub land	15.22	Built-up area	0
Other forest	21.19	Unused land	0.65

**Table 3:** Ecological resilience of different land-use types.

Land-Use Type	Resilience	Land-Use Type	Resilience
Urban area	0	Flood land	5
Rural residential area	1	River	6
Un-used land	2	Reservoir and lakes	7
Dry land	3	Grass land	8
Paddy land	4	Forestry	9

**Table 4:** Classification of coupling coordination degree of urbanization and ecological security.

Type	Coupling Coordination Degree	Subtype	Explanation
Advanced coordination	0.8<D≤1	Advanced coordination: urbanization lag	$E_{i}-U_{i}>0.1$
		Advanced coordination: ecological security lag	$U_{i}-E_{i}>0.1$
		Advanced coordination	$0\leq \left|U_{i}-E_{i}\right|\leq 1$
Basic coordination	0.5<D≤0.8	Basic coordination: urbanization lag	$E_{i}-U_{i}>0.1$
		Basic coordination: ecological security lag	$U_{i}-E_{i}>0.1$
		Basic coordination	$0\leq \left|U_{i}-E_{i}\right|\leq 1$
Basic un-coordination	0.3<D≤0.5	Basic un-coordination: urbanization blocked	$E_{i}-U_{i}>0.1$
		Basic un-coordination: ecological security blocked	$U_{i}-E_{i}>0.1$
		Basic un-coordination	$0\leq \left|U_{i}-E_{i}\right|\leq 1$
Serious un-coordination	0<D≤0.3	Serious un-coordination: urbanization blocked	$E_{i}-U_{i}>0.1$
		Serious un-coordination: ecological security blocked	$U_{i}-E_{i}>0.1$
		Basic un-coordination	$0\leq \left|U_{i}-E_{i}\right|\leq 1$

**Table 5:** The stability test results of urbanization and ecological security.

Variables ^1^	ADF T-Statistics	Critical Value at Significant Level			Results
		5%	10%	5%	
DlnU of Wuhan city	−4.4253	−4.2970	−3.2126	−2.7476	Stationarity
DlnE of Wuhan city	−3.0452	−4.2000	−3.1753	−2.7289	Stationarity
DlnU of Ezhou city	−3.2276	−4.2000	−3.1753	−2.7289	Stationarity
DlnE of Ezhou city	−3.0403	−4.2000	−3.1753	−2.7289	Stationarity
DlnU of Huanggang city	−2.7414	−4.2000	−3.1753	−2.7289	Stationarity
DlnE of Huanggang city	−3.0247	−4.2000	−3.1753	−2.7289	Stationarity
DlnU of Huangshi city	−3.4067	−4.2000	−3.1753	−2.7289	Stationarity
DlnE of Huangshi city	−3.1548	−4.2000	−3.1753	−2.7289	Stationarity
DlnU of Xianning city	−2.8865	−4.2000	−3.1753	−2.7289	Stationarity
DlnE of Xianning city	−3.5927	−4.2000	−3.1753	−2.7289	Stationarity
DlnU of Xiaogan city	−3.5427	−4.2000	−3.1753	−2.7289	Stationarity
DlnE of Xiaogan city	−3.0515	−4.2000	−3.1753	−2.7289	Stationarity
DlnU of Xiantao city	−3.1562	−4.2000	−3.1753	−2.7289	Stationarity
DlnE of Xiantao city	−3.0085	−4.2000	−3.1753	−2.7289	Stationarity
DlnU of Qianjiang city	−4.9535	−4.2970	−3.2126	−2.7476	Stationarity
DlnE of Qianjiang city	−3.3523	−4.2000	−3.1753	−2.7289	Stationarity
DlnU of Tianmen city	−3.3268	−4.2000	−3.1753	−2.7289	Stationarity
DlnE of Tianmen city	−3.3887	−4.2000	−3.1753	−2.7289	Stationarity

^1^ DlnU represents the log value of urbanization quality after first-order difference; DlnE represents the log value of ecological security level after first-order difference.

## Data Availability

Not applicable.
